# Fragmentation of CagA Reduces Hummingbird Phenotype Induction by *Helicobactor pylori*

**DOI:** 10.1371/journal.pone.0150061

**Published:** 2016-03-02

**Authors:** Chih-Chi Chang, Wein-Shung Kuo, Ying-Chieh Chen, Chin-Lin Perng, Hwai-Jeng Lin, Yueh-Hsing Ou

**Affiliations:** 1 Department of Biotechnology and Laboratory Science in Medicine, School of Biomedical Science and Engineering, National Yang-Ming University, Taipei, Taiwan; 2 Intensive Care Unit, Cheng-Hsin General Hospital, Taipei, Taiwan; 3 Division of Digestive Medicine, Taipei City Hospital Yangming Branch, Taipei, Taiwan; 4 Division of Gastroenterology, Department of Medicine, VGH-Taipei, Taiwan, and School of Medicine, National Yang-Ming University, Taipei, Taiwan; 5 Division of Gastroenterology and Hepatology, Department of Internal Medicine, Taipei Medical University, Shuang-Ho Hospital, New Taipei City, Taiwan, and Division of Gastroenterology and Hepatology, Department of Internal Medicine, School of Medicine, College of Medicine, Taipei Medical University, Taipei, Taiwan; Jawaharlal Nehru University, INDIA

## Abstract

Infection with *Helicobacter pylori (H*. *pylori)* has been linked to various gastro-intestinal diseases; nevertheless it remains to be clarified why only a minority of infected individuals develop illness. Studies from the West have indicated that the *cagA* gene and the associated EPIYA genotype of *H*. *pylori* is closely linked to the development of severe gastritis and gastric carcinoma; however, as yet no consistent correlation has been found among the bacteria from East Asia. In addition to genotype variation, the CagA protein undergoes fragmentation; however, the functional significance of fragmentation with respect to *H*. *pylori* infection remains unknown. In this study, we isolated 594 *H*. *pylori* colonies from 99 patients and examined the fragmentation patterns of CagA protein using immunoblotting. By analyzing the ability of the isolates to induce the host cell morphological transition to the highly invasive hummingbird phenotype, we demonstrated that *H*. *pylori* colonies with substantial CagA fragmentation are less potent in terms of causing this morphological transition. Our results uncovered a functional role for CagA fragmentation with respect to *H*. *pylori*-induced hummingbird phenotype formation and these findings suggest the possibility that the post-translational processing of CagA may be involved in *H*. *pylori* infection pathogenesis.

## Introduction

*Helicobacter pylori* (*H*. *pylori*) is a common human pathogen that has been closely linked to various gastric diseases, including chronic gastritis, peptic ulcers, gastric cancer and MALT-lymphoma [1–4]. Infection with *H*. *pylori* mostly through person-to-person transmission, but also occurs via contaminated foods [5–7]. The bacterial pathogen colonizes the stomach in manner whereby it is found selectively near or on the epithelial cell layer [6, 8]. *H*. *pylori* infection is found in more than half of the world’s population; nevertheless the outcome of this infection varies significantly among individuals. While most show no symptoms, some develop chronic gastritis, gastric ulcers, or even gastric cancer [9]. Gastric cancer is the fifth most common cancer worldwide and its prevalence is especially high in East Asia.

The mechanisms linking *H*. *pylori* infection to the development of gastric diseases have attracted a great deal of interest recently. There is ample evidence indicating that host susceptibility, environmental factors, and bacteria strain variability all contribute to the pathogenesis of *H*. *pylori* infections [2, 10–13]. The *H*. *pylori* genome encodes about 1,500 proteins and most disease-causing *H*. *pylori* strains have been shown to possess the *cag* pathogenicity island (*cag* PAI) [14–16], a 40-kb DNA segment that harbors cytotoxin-associated gene A (*cagA*) at its 3’ terminal. The *cag* PAI contains a variable number of genes, which can range from 26 to 32 [17, 18]; these include genes that encode proteins that can form a type IV secretion system (TFSS) as well as CagA. It has been shown that the *cag* PAI is present in 50% to 60% of *H*. *pylori* strains isolated in Western countries, but is found in more than 90% of strains isolated in East Asia [15, 19, 20]. A number of studies have indicated that when a *H*. *pylori* strain harbors the *cagA* gene, this significantly increases the risk of developing severe gastritis and gastric carcinoma [21–25].

The molecular weight of the CagA protein ranges between 120 kDa to 145 kDa, depending on the number of the 34-amino acid repeats present at its C-terminal; these consists of a conserved pentapeptide Glu-Pro-Ile-Tyr-Ala (EPIYA). EPIYA can be further classified into four distinct genotypes, EPIYA-A, EPIYA-B, EPIYA-C and EPIYA-D, based on their surrounding sequences [26, 27]. Most Western *H*. *pylori* isolates contain only one EPIYA-C motif (EPIYA-ABC genotype), while some have two or three EPIYA-C repeats (EPIYA-ABCC and EPIYA-ABCCC). Previous studies have indicated that *H*. *pylori* strains carrying more EPIYA-C motifs are more closely associated with gastric cancer [28–30]. In contrast, *H*. *pylori* isolates from East-Asian countries are mostly of the EPIYA-ABD genotype [31–33]. While a number of studies indicated that there is no consistent association between ABD genotype and disease outcomes [31, 34–36], Johns et al have analyzed the Korean strains of *H*. *pylori* and found a statistical link between CagA-ABD and gastric cancer [37].

After *H*. *pylori* infection, CagA enters the host cells via the TFSS-mediated pathway [38–40] where it is subjected to phosphorylation at the EPIYA motif by the Abl and Src family tyrosine kinases (SFKs) [41–44]. It has been shown that phosphorylation at different EPIYA motifs recruits different signaling molecules to the plasma membrane, resulting in modulation of multiple signaling pathways. The tyrosine phosphorylated EPIYA-A or B forms binary complex with kinase Csk, which then phosphorylates and inactivates SFK, preventing Cag A from further phosphorylation and activation of its downstream components [45, 46]. Phosphorylation at EPIYA-C or D activates SH2-containing phosphatase (SHP-2) [27, 47], resulting in the activation of MAPK signaling and inactivation of focal adhesion kinase (FAK). FAK inhibition causes the infected cells to assume an elongated cell shape [48] which could be shown in an in vitro assay of the *H*. *pylori* infected cells. This morphology is known as the hummingbird phenotype. Membrane tethering and activation of SHP-2 by the tyrosine-phosphorylated CagA is necessary and sufficient for the induction of the hummingbird phenotype [48, 49], which may be involved in malignant transformation [29, 50]. It has been shown that *H*. *pylori* strains possessing more EPIYA-C repeats undergo more extensive CagA phosphorylation and this causes a greater degree of morphological transition among the infected cells [48, 49].

In addition to the 135 kDa full-length CagA (p135^CagA^), the protein can be further processed to form a fragment of about 100 kDa (p100^CagA^) [51–53]. Interestingly, fragmentation of p135^CagA^, though frequently found among East Asia *H*. *pylori* strains, is rare among strains from the West [51–53]. The influence of CagA size variation on host responses has received little attention. In this study, we characterized the fragmentation patterns of CagA protein from 96 *H*. *pylori* colonies with the EPIYA-ABD genotype that were isolated from 99 gastric patients resident in Taiwan. We evaluated the effect that fragmentation had on the morphological transition of infected cells by analyzing hummingbird phenotype formation. In this study we were able to show that the ability of *H*. *pylori* to induce the hummingbird phenotype is significantly reduced when there is extensive CagA fragmentation. To the best of our knowledge, this is the first report linking CagA fragmentation to the formation of the *H*. *pylori*-mediated hummingbird phenotype. Our findings point to the possibility that post-translational processing of CagA may be involved in *H*. *pylori* infection pathogenesis.

## Materials and Methods

### Tissue specimens

Biopsy specimens from the gastric antrum were collected from 99 Taiwanese patients, all have provided their written consent to participate in this study. These consisted of 20 samples from gastric cancer (GC) patients, 32 samples from gastric ulcer (GU) patients, 31 samples from duodenal ulcer (DU) patients and 16 samples from chronic gastritis (CG) patients (Table 1). The study was approved by the Institutional Review Board, Taipei Veterans General Hospital (VGH) and Institutional Review Board of National Yang-Ming University; samples were obtained by the Division of Gastroenterology, Department of Medicine, VGH Taipei.

**Table 1:** Clinical characteristics of the gastric patients in this study.

Disease	Number of patients (M/F)*^a^*	Age range in years	Mean age in years (M/F)*^a^*
Gastric cancer (GC)	20 (14/6)	44–89	64.3 (70.5/58.2)
Gastric ulcers (GU)	32 (26/6)	35–82	62.6 (66.9/58.3)
Duodenal ulcers (DU)	31 (15/16)	23–83	53.5 (54.8/52.3)
Chronic gastritis (CG)	16 (10/6)	15–91	54.1 (59.6/48.5)
Total	99 (65/34)	15–91	58.6 (63.0/54.3)

^*a*^M/F, Male/Female.

All patients under study are free from using antibiotics or proton pump inhibitors. Patients with the following conditions were excluded from this study: a tendency to bleed (platelet count less than 50,000/mm^3^, a prothrombin time prolonged more for than 3 seconds or taking anticoagulants), lactating or pregnant women, the inability to give informed consent, the inability to cooperate with an operation on the UGI tract and severe cardiovascular, hepatic or renal disease.

### Isolation of *H*. *pylori* from the tissue specimens

The biopsy specimens were inoculated onto brain-heart infusion agar plates supplemented with 10% sheep blood that contained nalidixic acid (10 μg/ml), trimethoprim (5 μg/ml), vancomycin (3 μg/ml) and amphotericin (2 μg/ml). All plates were incubated at 37°C for up to 5 days under microaerobic conditions (12% CO_2_). Six single colonies were then selected from each primary culture plate and inoculated onto six fresh brain-heart infusion agar plates without antibiotics. The bacterial lawn that grew up on these plates were collected and chromosomal DNA was extracted from the collected bacteria by QIAamp DNA Mini Kit (Qiagen). The purified DNA was stored at -80°C until PCR analysis. Two strains, *H*. *pylori* 26695 and *H*. *pylori* 60190, were used as standard strains for comparison with the new isolates.

### Analysis by PCR of the 5’-end and the 3’-end regions of *cagA*, the latter encoding the EPIYA motif of CagA

The 5’-region and the 3’ variable region of *cagA*, the latter encoding the EPIYA motif of CagA, were amplified by polymerase chain reactions (PCR) using specific primers. The primers were forward, cagA-F: 5’-GATAACAGGCAAGCTTTTGAGGGA-3’, and reverse, cagA-R: 5’-CCATGAATTTTTGATCCGTTCGG-3’ for the *cagA* 5’ region, and forward, EPIYA-F: 5’-ACCCTAGTCGGTAATGGGTTA-3’ and reverse, EPIYA-R: 5’-CATCAATCGTAGCGTAAATGG-3’ for the *cagA* 3’ EPIYA region. The PCRs were carried out using 20 μl reaction mixtures containing 2 μl of genomic DNA (0.4 μg), 2 μl of each primer (2 μM), 2 μl of 10X reaction buffer, 2 μl of 2.5 mM dNTP, 0.5 μl Taq DNA polymerase and 9.5 μl sterilized water using a GeneAmp 2400 PCR system. After heating to 94°C for 90 s, amplification (94°C for 60 s, 54°C for 60 s, and 72°C for 60 s) was carried out for 25 cycles. The mixture was then held at 72°C for 5 min to complete the elongation step. The PCR products were separated by electrophoresis on 2% agarose gels and then examined under UV illumination. The PCR products of the *cagA* 3’-end region were further purified using a QIA quick gel extraction kit (Qiagen, Valencia, CA) followed by sequenced via a custom sequencing service (Mission Biotech, Taipei, Taiwan). The DNA sequences were aligned and analyzed using appropriate software (DNA Start).

### Analysis of the CagA protein fragmentation and phosphorylation by immunoblotting

For CagA protein fragmentation analysis, *H*. *pylori* isolates were collected and lysed in lysis buffer (50 mM Tris-HCl, pH 7.5, containing 100 mM NaCl, 5 mM EDTA, 1% Brij-35) for 10 min on ice. For analysis of CagA phosphorylation, *H*.*pylori*-infected AGS cells were washed with PBS and resuspended in 400 μl lysis buffer (50 mM Tris-HCl pH 7.4, 0.1% saponin, 1 mM ortho-vanadate, containing complete protease inhibitors) for 10 min at 4°C. The lysates were centrifuged at 14,000 rpm for 5 min at 4°C, and supernatants were transferred to a new 1.5 ml tube. The concentration of protein in each sample was determined by the Bradford assay (Pierce). The proteins (20 μg) were separated by SDS-PAGE (7.5% gel) and transferred to a PVDF membrane (Roche) for immunoblotting. The membrane was soaked in blocking buffer (50 mM Tris-HCl, pH 7.5, 150 mM NaCl, 5% milk) for 1 h, amounts of CagA and phosphorylated-CagA were determined by reacting with polyclonal anti-CagA antibody and monoclonal anti-phosphotyrosine antibody PY99 from Santa Cruz, respectively. The membranes were treated with either goat anti-rabbit or anti-mouse IgG conjugated with horseradish peroxidase (Santa Cruz). Immunoreactive bands were visualized by Western Lightning Chemiluminescence Reagent Plus (PerkinElmer Life Sciences) and analyzed using a luminescent image analyzer (GE LAS-4000).

### Cell culture and *H*. *pylori* infection

For *H*. *pylori*-induced hummingbird phenotype analysis, AGS cells were cultured in RPMI 1640 medium containing 10% FBS. Each of the *H*. *pylori* isolates were suspended in RPMI 1640 and this suspension was used for AGS cell infection at a multiplicity of infection (MOI) of 100 for 5 h. The morphology of the AGS cells was determined using a microscope, and cells showing an elongated hummingbird phenotype (a ratio of the longest protrusion to the shortest diameter of greater than 2) were scored in ten different fields (10 samples at x40 magnification). AGS cells (ATCC CRL 1739) were purchased from Food Industry Research and Development Institute (FIRDI), Taiwan, in 2002.

### Mass spectrometry

The *H*. *pylori*-88 (type-3) bacteria were washed with cold PBS and resuspended in 1 ml lysis buffer (50 mM Tris-HCl pH 7.4, 150 mM NaCl, 1% Triton X-100, 0.25% sodium-deoxycholate, 1 mM EDTA pH 8.0, and 1 mM ortho-vanadate), in the presence of complete protease inhibitor (Roche) for 30 min at 4°C. Three μg anti-CagA polyclonal antibody (b-300 Santa Cruz Biotechnology) were added to the supernatant and incubated overnight at 4°C. Immune complexes were precipitated by the addition of protein G–Sepharose (50μl) for 2 h and washed 3 times with lysis buffer. Finally, the mixture was boiled for 5 min at 95°C in SDS buffer, and separated by SDS-PAGE. The Coomassie Blue-stained bands corresponding to molecular weight of 100and 110 kD were excised from gel and subjected to in-gel digestion with Asp-N. The peptide mixture was mixed (1:1) with a saturated solution of a-cyano-4-hydroxycinnamic acid in 33% acetonitrile–0.1% trifluoroacetate, separated by reverse phase chromatography and directly applied to MALT-TOF mass spectrometer (Autoflex-T1; Bruker Daltonics Inc., Bellerica, MA, USA). The mass spectrum data was aligned with *H*. *pylori*-88 CagA protein sequence.

### Statistics

All data were analyzed using SPSS 20.0 (SPSS Inc.). Chi-square tests or Fisher’s exact tests were used to analyze statistical significance when comparing the *cagA* EPIYA genotype, CagA protein pattern and the disease types. A *p* value of < 0.05 was considered to be statistically significant. Associations between the CagA protein pattern and hummingbird phenotype induction were analyzed by Kruskal-Wallis test with multiple pairwise comparisons by using Mann-Whitney U test. The Bonferroni method was applied for post-hoc comparisons.

## Results

### Isolation of *H*. *pylori* colonies and analysis of the size variation of the *cagA* gene

**Fig 1:**
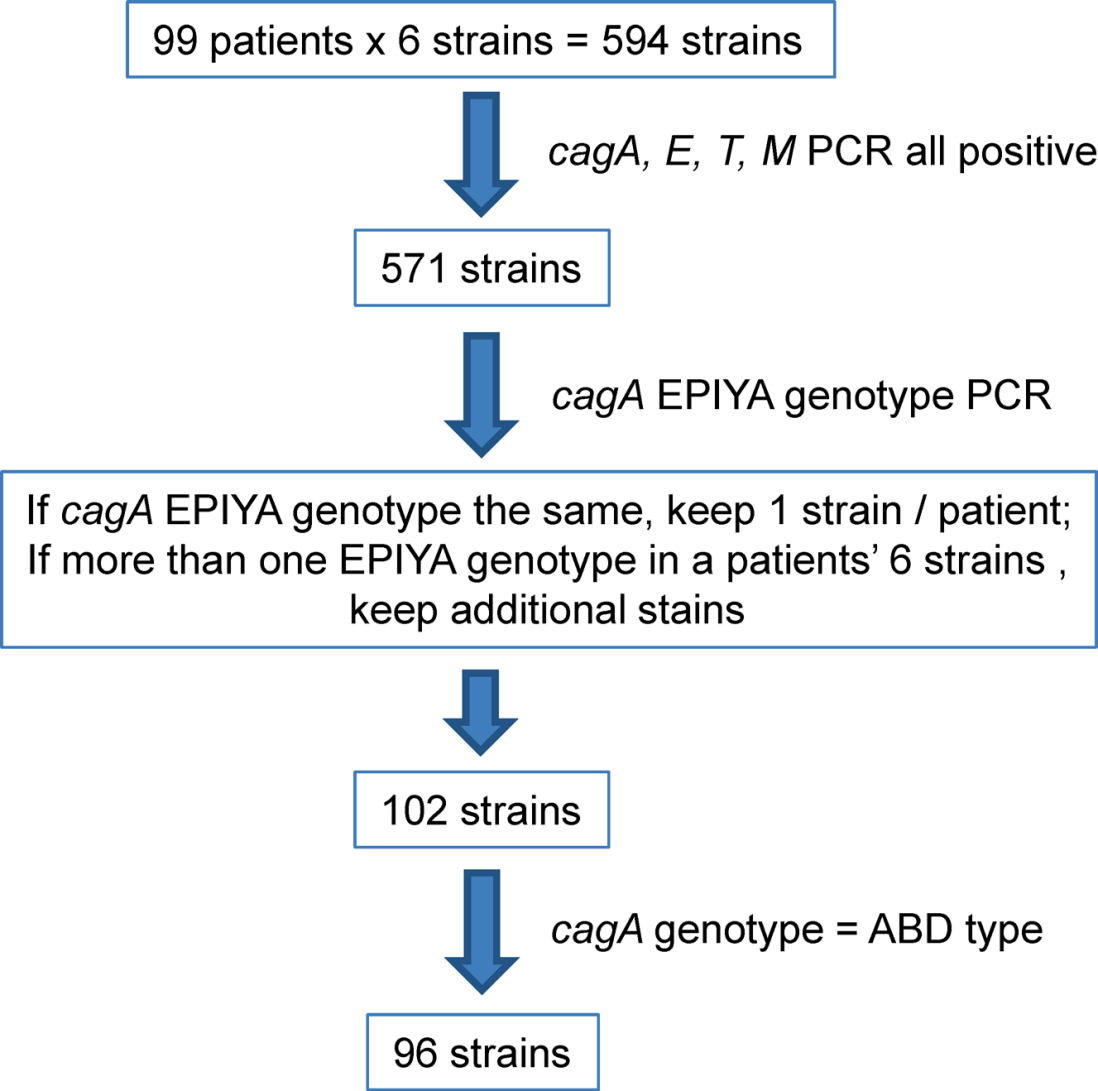
Flow chart showing the selection of the *H*. *pylori* colonies.

**Fig 2:**
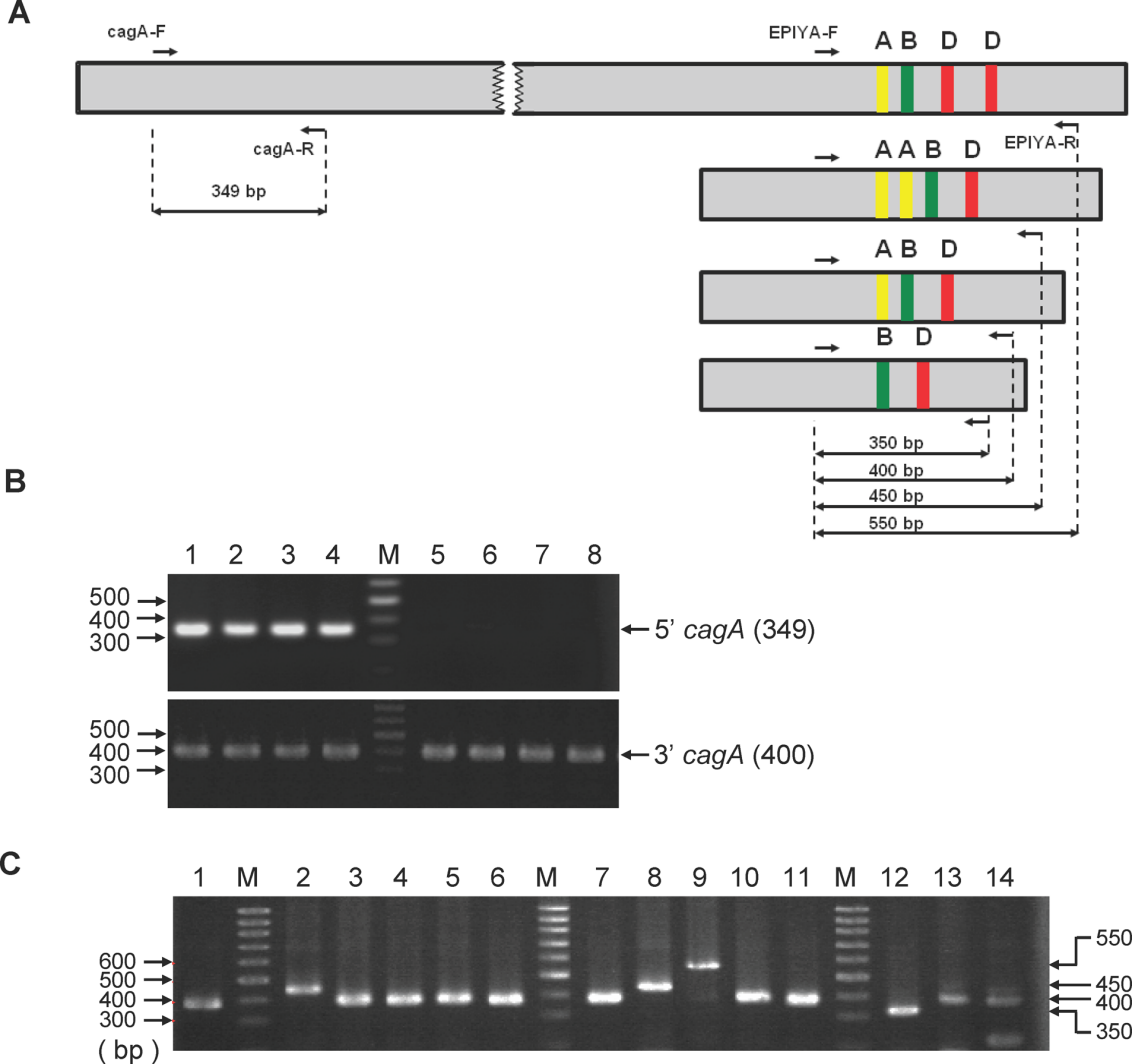
PCR amplification of the *cagA* 5’ constant region and the 3’ variable region. (A) Schematic representation of the relative primer site of the *cagA* gene (small arrows) used in this study, and the expected sizes (long arrows) of the amplified specific EPIYA motif products. (B) Genomic DNA from *H*. *pylori* colonies was used as templates to amplify the 5’ (above) and 3’ (below) ends of the *cagA* gene by PCR using the gene specific primers as described in the Materials and Methods. PCR products were separated by electrophoresis using 2% agarose gels that were stained with ethidium bromide. Lanes are: M, 100-bp DNA markers. 1, GC 25–1; 2, GC 68–1; 3, GU 71–1; 4, GU 94–1; 5, GC 25–5; 6, GC 68–6; 7, GU 71–2; and 8, GU 94–3. Shown on the right are the sizes of the PCR products in base pairs. (C) PCR analysis of the *cagA* 3’ region expressing variable number of EPIYA motifs. Lanes are: M: 100-bp DNA markers. 1, *H*. *pylori* 26695; 2, GC 6–1; 3, GC 6–2; 4, GC 7–1; 5, GC 7–2; 6, GC 7–3, 7, GC 11–1; 8, GU 12–1; 9: GU 13–1; 10, GU 13–2; 11, CG 14–1; 12, GC 21–3; 13: DU 47–3; and 14, DU 64–3. Shown on the right are the sizes in base pairs.

To characterize *cagA* gene variation, we isolated *H*. *pylori* colonies from the biopsy specimens of 99 patients with various gastric diseases (Table 1). Six single colonies were selected for each patient, giving a total of 594 colonies. Characterization of the *H*. *pylori* isolates was carried out according to the flow chart shown in Fig 1. The 5’-end highly conserved region and the 3'-end variable region, the latter expressing the EPIYA motif, were amplified by PCR using the specific primers as shown in Fig 2A. As shown in Fig 2B, PCR products of 349 bp and 400 bp, representing the 5’-end and 3’-end fragments, respectively, were observed. The *cagA* 5'-end was present in 590 of the 594 *H*. *pylori* isolates (99.3%) and thus was absent only in four colonies (GC 25–5, GC 68–6, GU 71–2 and GU 94–3). All *H*. *pylori* isolates were found to contain the *cagA* 3'-end region expressing various EPIYA motifs. Further PCR analysis was performed on the *cagA* region expressing the variable number of EPIYA motifs. As shown in Fig 2C, four distinct PCR products, 350 bp, 400 bp, 450 bp, and 550bp in size, were obtained from the various *H*. *pylori* isolates. These results indicate that the 3'-end *cagA* regions of the various CagA proteins consisted of different repeats of the EPIYA motif. As a control, PCR analysis was also performed on the *cag* PAI region that encodes *cagE*, *cagT and cagM* in order to evaluate the integrity of the TFSS secretion apparatus. Amplifications of the *cagE*, *cagT*, *cagM* were carried out with the gene specific primers shown in S1 Table. Our results indicated that out of the 594 colonies, 571 were found to harbor all four *cag* PAI genes (S2 Table).

### Polymorphism of *cagA* 3’-region expressing the EPIYA motifs

**Fig 3:**
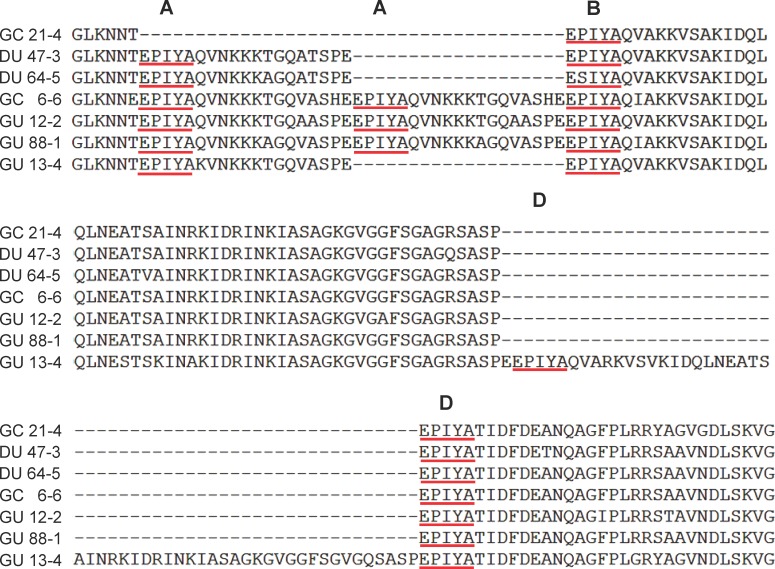
Amino acid sequence alignment of CagA EPIYA motifs. Alignment of the amino acid sequences of the CagA EPIYA motifs of the various *H*. *pylori* isolates. The amino acid sequences were derived from the DNA sequencing data of the PCR products using DNA Star software. The results indicate the GU 13–4 is of the EPIYA-ABDD genotype, that GC 6–6, GU12-2 and GU 88–1 are of the EPIYA-AABD type, that DU 47–3 and DU 64–5 belong to the EPIYA-ABD type, and that GC 21–4 is of the EPIYA-BD type. The positions of the EPIYA-A, EPIYA-B, and EPIYA-D motifs are indicated.

We next determined the DNA sequences of the region of *cagA* expressing the EPIYA motifs present in the isolated *H*. *pylori* colonies. The sequences of the EPIYA motifs were determined for the isolates from each patient. If the PCR products of all six isolates from one patient had exhibit similar size patterns, only one was randomly selected for further sequencing. In cases where two different size patterns of PCR products were found (patient number 6, 13, and 82), two appropriate colonies for each patient were selected for sequencing. Thus a total of 102 *H*. *pylori* colonies were chosen for further sequence analysis. A comparison of the results indicated that the EPIYA genotypes of the 350 bp, 400 bp, 450 bp and 550 bp amplicons were those of the BD, ABD, AABD, and ABDD types, respectively (Fig 3). The distributions of the *cagA* EPIYA genotypes across the *H*. *pylori* isolates for all four gastric diseases, including gastric cancer (GC), gastric ulcer (GU), duodenal ulcer (DU), and chronic gastritis (CG), are shown in Table 2. It is obvious that the majority of the *cagA* EPIYA in this study belong to the ABD genotype (96/102, 94.1%) and that this is followed in a decreasing order by AABD (2.9%), ABDD (2.0%) and BD (1.0%). A statistical analysis revealed no significant correlation between the disease outcome and the genotype of the *cagA* EPIYA (*p* = 0.359). Among the biopsy samples analyzed, three were shown to possess more than one EPIYA genotype isolates. These are: patient GC-6 (1 ABD and 5 AABD), patient GU-13 (3 ABD and 3ABDD), and patient CG-82 (5 ABD and 1 ABDD).

**Table 2:** The *cagA* EPIYA genotype distributions across the *H*. *pylori* isolates from the various clinical disease groups.

Genotype	Number of isolates (%)				
	GC (N = 21)	GU (N = 33)	DU (N = 31)	CG (N = 17)	Total (N = 102)
**ABDD**	**0 (0)**	**1 (3.0)**	**0 (0)**	**1 (5.9)**	**2 (2.0)**
**AABD**	**1 (4.8)**	**2 (6.1)**	**0 (0)**	**0 (0)**	**3 (2.9)**
**ABD**	**19 (90.4)**	**30 (90.9)**	**31 (100)**	**16 (94.1)**	**96 (94.1)**
**BD**	**1 (4.8)**	**0 (0)**	**0 (0)**	**0 (0)**	**1 (1.0)**

Abbreviations: GC, gastric cancer; GU, gastric ulcer; DU, duodenal ulcer; CG, chronic gastritis. N represents the number of bacterial isolates associated with this part of the study.

### Characterization of the CagA protein degradation patterns of the *H*. *pylori* colonies

**Fig 4:**
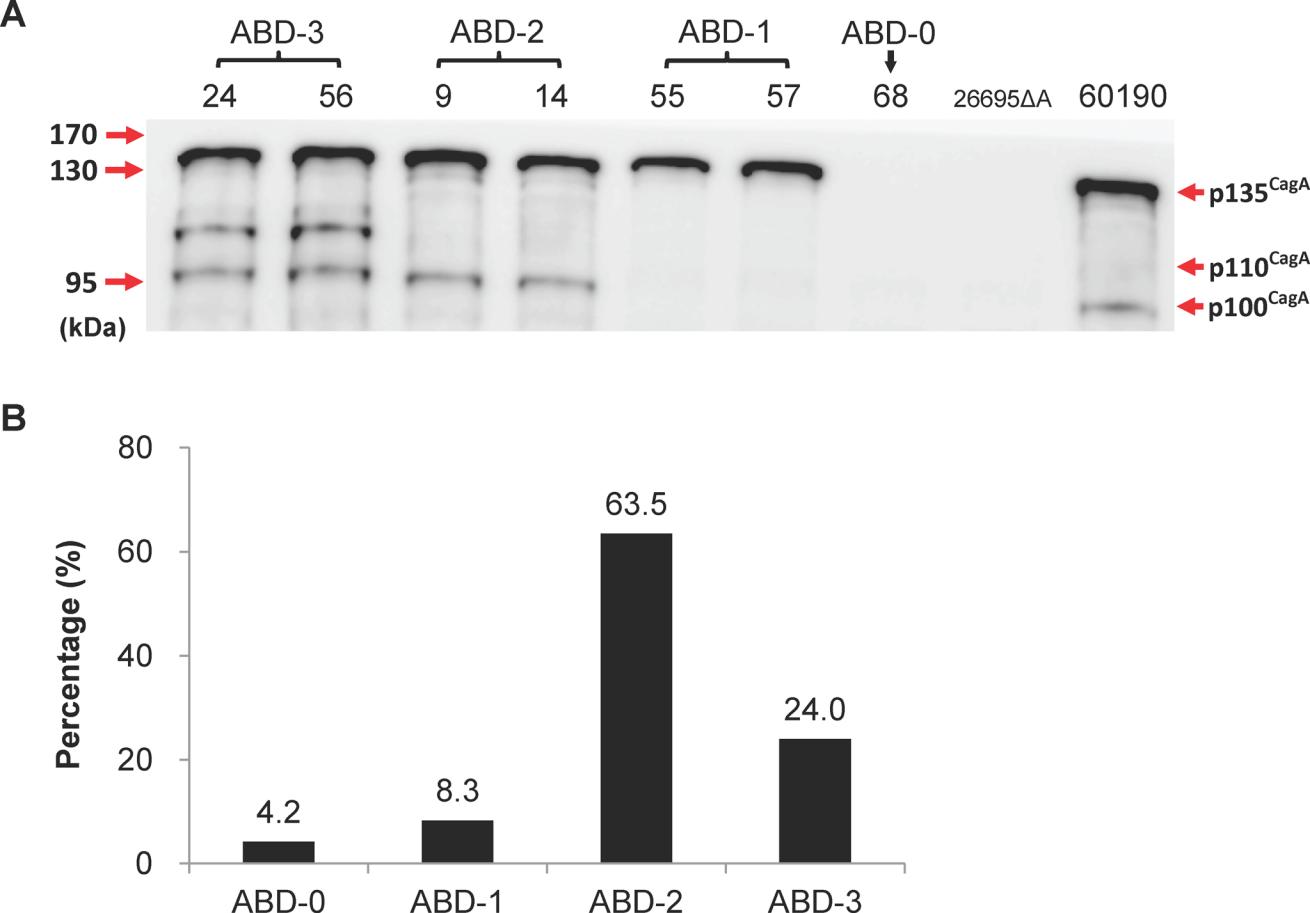
Analysis of the CagA protein patterns by immunoblot analysis. (A) Lysates of *H*. *pylori* isolated from the tissue specimens of various gastric patients were prepared as described in the Materials and Methods. Aliquots of protein (20 μg) were separated by SDS-gel electrophoresis and CagA was detected by immunoblotting using polyclonal anti-CagA antibody b-300 (Santa Cruz). The *H*. *pylori* 60190 and 26695 *cagA* knockout mutant (26695ΔA) were included as a positive and negative control, respectively, for CagA expression. The numbers at the top identify the various *H*. *pylori* colonies, while those on the left indicate the molecular weight markers in kDa. It can be seen that there are four types of CagA fragmentation patterns. (B) The percentages of ABD subtypes based on the CagA protein fragmentation patterns across the 96 *H*. *pylori cagA* ABD colonies that were analyzed and clearly ABD-2 is the predominant type.

It has previously been shown that there exists more than one *cagA* gene products in *H*. *pylori* [51–53]. To examine if the expression pattern of the CagA protein differs in the various strains with the same genotype, we analyzed the CagA protein patterns across all 96 *H*. *pylori* colonies harboring the ABD genotype by immunoblot analysis. As shown in Fig 4A, in addition to the 135 kDa full-length CagA, two immune-reactive bands with molecular weights of 100 and 110 kDa could be detected in some *H*. *pylori* isolates. We showed that these fragments were derived from CagA by duplicating the immunoblotting analysis using two additional monoclonal anti-CagA antibodies, Santa Cruz A-10 and GeneText B818M (data not shown). Moreover, mass spectrometry was performed to further confirm that these fragments were indeed CagA proteins (S2 Fig).

A summary of the immunoblotting results are presented in Fig 4B and Table 3. Four CagA protein expression patterns were found. Among the 96 *H*. *pylori* isolates analyzed, four (4.2%) did not exhibit any immune-reactive CagA bands (termed the ABD-0 type), whereas eight (8.3%) expressed only the 135 kDa full-length CagA (termed the ABD-1 type). The most prevalent CagA protein pattern consisted of two protein bands with molecular weights of 135 and 100 kDa (the ABD-2 type, 63.5%). Finally, about 24% of the isolates had three immune-reactive bands (termed the ABD-3 type), these bands had molecular weights of 135, 110 and 100 kDa.

**Table 3:** Distribution of CagA protein patterns across the *H*. *pylori* isolates from various clinical disease groups.

CagA pattern	Number of isolates (%)				
	GC (N = 19)	GU (N = 30)	DU (N = 31)	CG (N = 16)	total (N = 96)
**ABD-0**	**2 (10.5)**	**0 (0)**	**1 (3.2)**	**1 (6.3)**	**4 (4.2)**
**ABD-1**	**2 (10.5)**	**2 (6.7)**	**3 (9.7)**	**1 (6.3)**	**8 (8.3)**
**ABD-2**	**6 (31.6)**	**24 (80.0)**	**19 (61.3)**	**12 (75.0)**	**61 (63.5)**
**ABD-3**	**9 (47.4)**	**4 (13.3)**	**8 (25.8)**	**2 (12.5)**	**23 (24.0)**

Abbreviation: GC, gastric; GU, gastric ulcer; DU, duodenal ulcer; CG, chronic gastritis. N represents the number of bacterial isolates associated with this part of the study.

As can be noted in Table 3, the distributions of CagA protein patterns among the 19 *H*. *pylori* isolates from the gastric cancer patients were: ABD-0 (2, 10.5%), ABD-1 (2, 10.5%), ABD-2 (6, 31.6%), and ABD-3 (9, 47.4%). Statistical analysis indicates that the distribution of the CagA fragmentation patterns in the gastric cancer patients was significantly different from those of the non-cancer (DU, GU and CG) groups (*p* = 0.045). Further analysis by the Bonferroni correction method showed that the distribution of ABD-2 in gastric cancer group was significantly lower and that ABD-3 was significantly higher in the gastric cancer group than in the ulcer groups or the chronic gastritis group (*p*<0.05).

### CagA protein fragmentation reduces hummingbird phenotype induction by *H*. *pylori*

**Fig 5:**
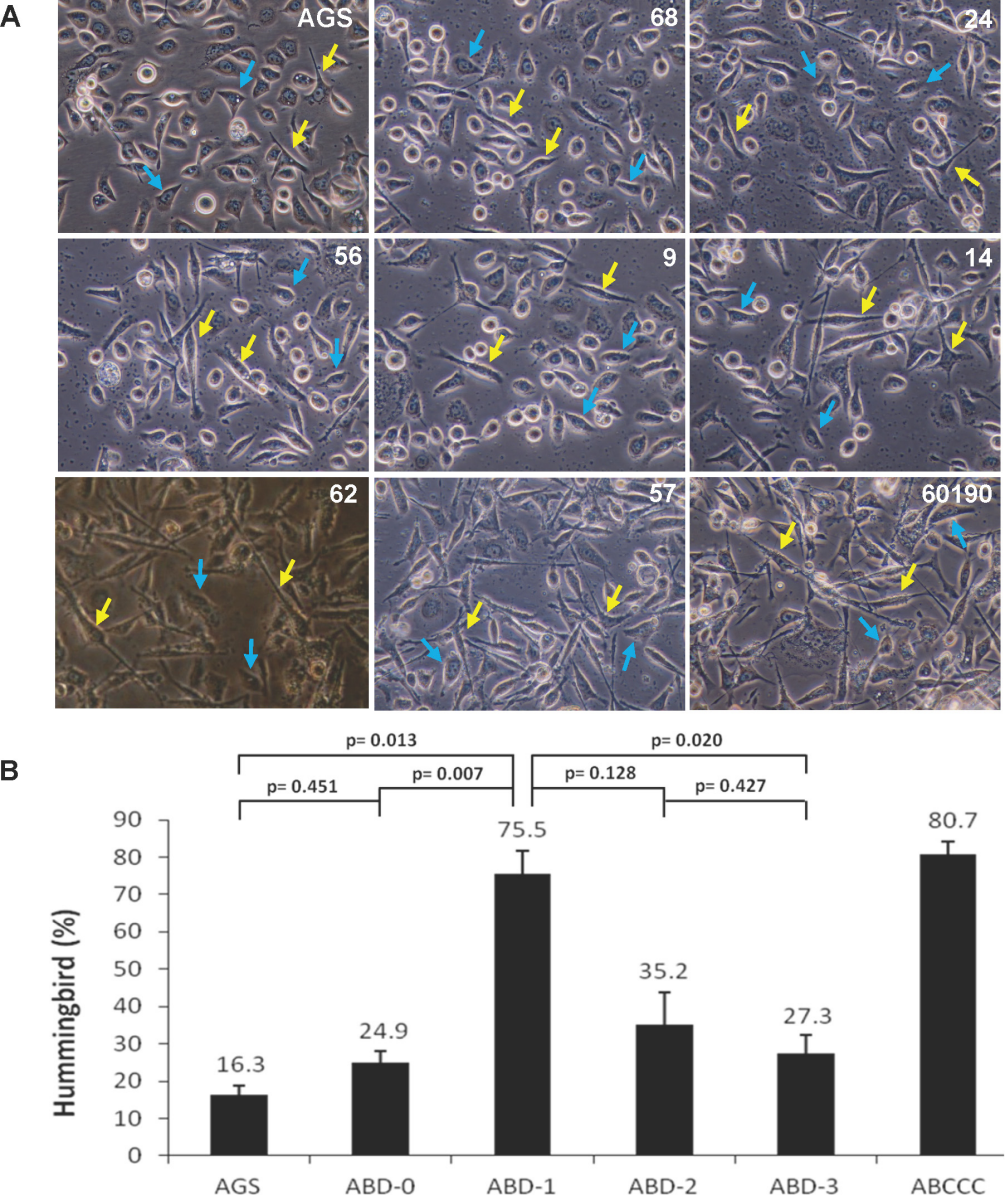
Effects of CagA protein fragmentation of *H*. *pylori* on the formation of hummingbird phenotype. (A) AGS cells were infected without (AGS) or with various subtypes of ABD-*H*. *pylori* at a MOI of 100 for 5 h. Cells showing the hummingbird phenotype were scored by microscopy. The *H*. *pylori* 60190 strain harboring the EPIYA-ABCCC genotype was included as a positive control. Yellow arrows indicate cells with the elongated hummingbird phenotype. Blue arrows are cells not undergoing morphological transitions. (B) Quantification of the results obtained in A. The percentages of cells with the hummingbird phenotype were calculated from the results of three independent experiments, each with 10 randomly selected fields. No significant difference was found between ABD-0 and the AGS cell only control.

Previous studies have indicated that phosphorylation on the EPIYA motifs of CagA is responsible for cytoskeletal rearrangements in the host cells. We next analyzed the hummingbird induction ability by the ABD type *H*. *pylori* colonies expressing the various CagA protein patterns. For each CagA protein pattern, four *H*. *pylori* colonies were used and thus a total of 16 *H*. *pylori* isolates were randomly selected for this assay. The AGS cells were infected with *H*. *pylori*, and the cells that acquired the hummingbird phenotype were counted. As can be seen in Fig 5A, the *H*. *pylori* isolates expressing only the full-length 135 kDa CagA (pattern ABD-1) were most potent at inducing the hummingbird phenotype (75.5%), which is higher when compared to morphological transitions induced by the bacteria with the ABD-2 (35.2%) or ABD-3 type (27.3%) (Fig 5B). It is of interest to note that, unexpectedly, the ABD-0 type, which expresses no detectable level of CagA, was also still capable of causing the hummingbird phenotype, which suggesting that there are factors other than CagA that may be involved in causing gastric cancer.

## Discussion

Infection with *H*. *pylori*, which is found in more than half of the world’s population, has been implicated in stomach-related diseases. The c*agA* is a virulence gene shown to have a high association with the pathogenesis caused by *H*. *pylori* infection [1, 3, 54]. Studies have indicated that the *H*. *pylori* strains with more EPIYA-C repeats in *cagA* are more prone to inducing gastric cancer [30–32]. In this study, we have characterized the *cagA* gene polymorphism of 102 *H*. *pylori* colonies isolated from 99 Taiwanese patients with various gastric diseases, including gastritis, ulcer and cancer. We showed that EPIYA-ABD is the predominant genotype (96/102, 94%) of the *H*. *pylori* isolates in Taiwan. The *cagA* EPIYA-C genotype, which is most frequent in Western strains, was absent from these Taiwanese isolates. Moreover, we did not detect any significant correlation between the EPIYA-ABD genotype and gastric cancer in these patients.

In addition to the *cagA* EPIYA genotypes, demographic differences between the *H*. *pylori* isolates from Eastern and Western populations could also be found when the fragmentation of the CagA protein was evaluated. While CagA cleavage is rare in Western isolates, we and others [51–53] have shown that most Asian *H*. *pylori* isolates undergo CagA fragmentation. However, because there is no consistent correlation between EPIYA-ABD genotype and the clinical outcomes of *H*. *pylori* infection [31, 34–37], we hypothesized that the reason behind bacteria-mediated carcinogenesis may lie in the post translational processing of CagA. Based on the fragmentation patterns, we further divided the ABD genotype into four phenotypic classes, ABD-0, ABD-1, ABD-2 and ABD-3. Significantly, we show that ABD-1, which expresses only the full-length CagA, is more potent when inducing the hummingbird phenotype compared to bacteria with no detectable CagA levels (ABD-0) or with expression of degraded CagA (ABD-2 and ABD-3). It is conceivable that degradation of CagA may reduce the amount of active protein involved in causing the morphological transition. However, we have noticed that the relative abundance of the full-length CagA in ABD subtypes varied from experiment to experiment, and ABD-3 and ABD-2 may have as much full length CagA compared to ABD-1. Interestingly, the morphological transition did not necessarily parallel with the full-length CagA levels. It could be that the amount of CagA entering the cells, and not that produced by the bacteria, is the determining factor of CagA functional expression. The C-terminal translocation signal of CagA, which is absent in both the 100 and 110 kDa CagA fragments, is required for the type IV-mediated translocation of the protein. It remains a possibility that these fragments may interfere with the full-length CagA translocation, and suppress its ability to cause morphological changes of the host cells. Consistent with this speculation, we have shown by *in vitro* infection assays that levels of CagA phosphorylation, occurring inside the host cells, were significantly higher in ABD-1, as compared to ABD-2 and ABD-3, infected cells (S3 Fig).

The finding that ABD-1 is most effective when causing the hummingbird phenotype leads to the suggestion that integrity of the CagA protein should favor carcinogenesis and thus the bacteria of the ABD-1 phenotype will be more likely to induce carcinogenesis. It is intriguing to note that the percentage of ABD-3, but not that of ABD-1, is significantly higher among the gastric cancer patients. The reason behind this discrepancy is currently unclear. In addition to elicit an oncogenic insult, CagA is involved in the stimulation of immune response [55]. We speculate that suppression of CagA function by fragmentation may at the same time diminish its capacity to stimulate the inflammatory response of the host cells [56]. Inflammation recruits infiltration of white blood cells, which could then result in gastritis; the symptoms of gastritis may then urge the infected individuals to seek medical help, which will reduce the opportunity for long term colonization with *H*. *pylori* and this in turn might decrease the chance of gastric cell transformation [55–57]. In this study we have shown by *in vitro* assays that *H*. *pylori* harboring the ABD-3 phenotype remains capable of inducing the hummingbird phenotype, albeit with a much less potency. It is plausible that under circumstances where there is reduced immunological activity, the bacteria harboring the CagA ABD-3 phenotype may be able to evade the surveillance system, allowing the CagA protein to exert a low but persistent signal that affects the host cells. Thus the infected individual may be asymptomatic for a long time, but ultimately may undergo carcinogenesis. It is worth mentioning that ABD-3 harbors a distinctive 110 kDa CagA fragment not found in the other ABD phenotypes and the possibility that this protein fragment may have a role in the gastric cancer progression warrants further investigation.

In summary, we have shown that the fragmentation patterns of CagA protein is relevant to the *H*. *pylori* infection-induced hummingbird phenotype formation. Our findings on the presence of a significantly high percentage of the CagA EPIYA ABD-3 phenotype among the *H*. *pylori* colonies from gastric cancer specimens provide important insights linking disease outcome to the *H*. *pylori* infection. A better understanding of the basis of CagA fragmentation and of the functional significance of the various CagA fragments will undoubtedly advance our knowledge of *H*. *pylori*-related gastric disorder in the future.

## Supporting Information

S1 Fig Analysis of the CagA protein patterns by immunoblotting. Lysates of *H*. *pylori* isolated from the tissue specimens of various gastric patients were prepared as described in the Materials and Methods. Aliquots of protein (20 μg) were separated by SDS-gel electrophoresis and CagA was detected by immunoblotting using polyclonal anti-CagA antibody b-300 (Santa Cruz). The *H*. *pylori* 60190 and 26695 *cagA* knockout mutant (26695ΔA) were included as a positive and negative control, respectively, for CagA expression. The numbers at the top identify the various *H*. *pylori* colonies, while those on the left indicate the molecular weight markers in kDa. It can be seen that there are four types of CagA fragmentation patterns. Urease α was used as loading control. (The uncropped immunoblot of CagA shows the entire fragmentation patterns.)
(TIFF)

S2 Fig Peptide mass fingerprinting of p100^CagA^and p110^CagA^. The bands of p100^CagA^ and p110^CagA^ from HP-88 were excised from the stained SDS-PAGE gel and subjected to in-gel digestion with Asp-N. The digested peptides were analyzed by mass spectrometry and mass spectrum data was aligned with HP-88 CagA protein sequence. More than 10 peptides were identified by MALDI-MS, all were identical in sequence to the HP-88 CagA protein. The complete HP-88 CagA protein sequence is shown, peptides obtained from MALDI-TOF MS were shown in red.
(TIF)

S3 Fig Analysis of CagA phosphorylation in infected cells. AGS cells were infected with various *H*. *pylori* isolates of the indicated genotypes. Total cell lysates of the infected cells were prepared as described in the Materials and Methods. Proteins (20 μg) were separated by SDS-gel electrophoresis, amounts of CagA and phosphorylated-CagA were determined by immunoblotting using polyclonal anti-CagA antibody b-300 (Santa Cruz) and anti-phosphotyrosine antibody PY99 (Santa Cruz). The *H*. *pylori* 60190 and 26695 *cagA* knockout mutant (26695ΔA) were included as a positive and negative control, respectively, for CagA expression. AGS was an uninfected cell control. Numbers at the top are *H*. *pylori* isolates of various ABD genotypes, molecular weight markers are shown in kDa on the right, and β-Actin was shown as a protein loading control.
(TIFF)

S1 Table Sequence information for primers. (TIFF)

S2 Table The *cag* gene status of *H*. *pylori* isolates from various clinical disease groups. (TIFF)
