# Supplementary figures and images for: Fragmentation of CagA Reduces Hummingbird Phenotype Induction by Helicobactor pylori
Source: PLoS One. 2016 Mar 2;11(3):e0150061. doi: 10.1371/journal.pone.0150061 (PMC4775065; doi:10.1371/journal.pone.0150061)

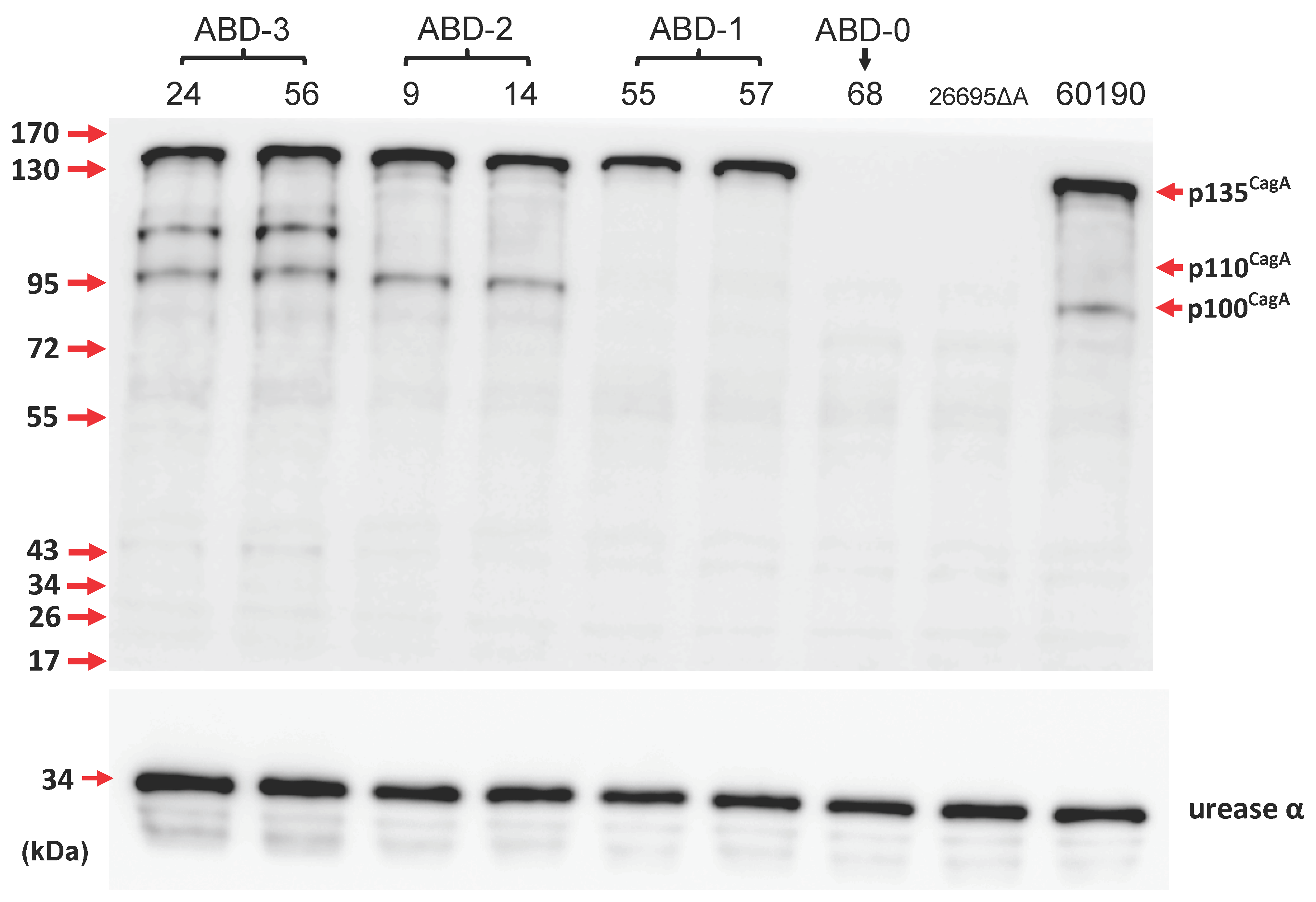

Supplement: S1 Fig — Lysates of H. pylori isolated from the tissue specimens of various gastric patients were prepared as described in the Materials and Methods. Aliquots of protein (20 μg) were separated by SDS-gel electrophoresis and CagA was detected by immunoblotting using polyclonal anti-CagA antibody b-300 (Santa Cruz). The H. pylori 60190 and 26695 cagA knockout mutant (26695ΔA) were included as a positive and negative control, respectively, for CagA expression. The numbers at the top identify the various H. pylori colonies, while those on the left indicate the molecular weight markers in kDa. It can be seen that there are four types of CagA fragmentation patterns. Urease α was used as loading control. (The uncropped immunoblot of CagA shows the entire fragmentation patterns.) (TIFF) [file pone.0150061.s001.tiff]

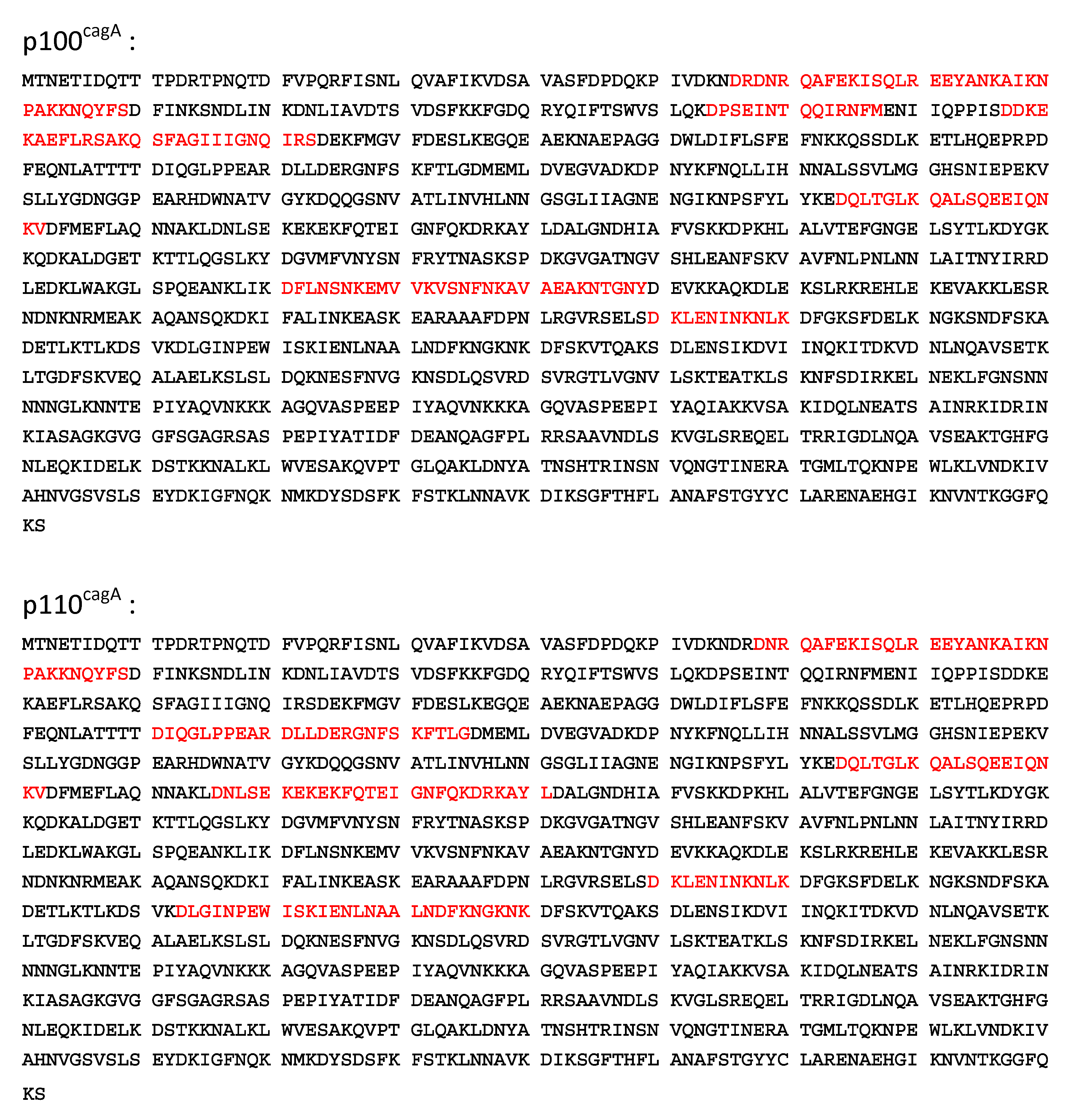

Supplement: S2 Fig — The bands of p100CagA and p110CagA from HP-88 were excised from the stained SDS-PAGE gel and subjected to in-gel digestion with Asp-N. The digested peptides were analyzed by mass spectrometry and mass spectrum data was aligned with HP-88 CagA protein sequence. More than 10 peptides were identified by MALDI-MS, all were identical in sequence to the HP-88 CagA protein. The complete HP-88 CagA protein sequence is shown, peptides obtained from MALDI-TOF MS were shown in red. (TIF) [file pone.0150061.s002.tif]

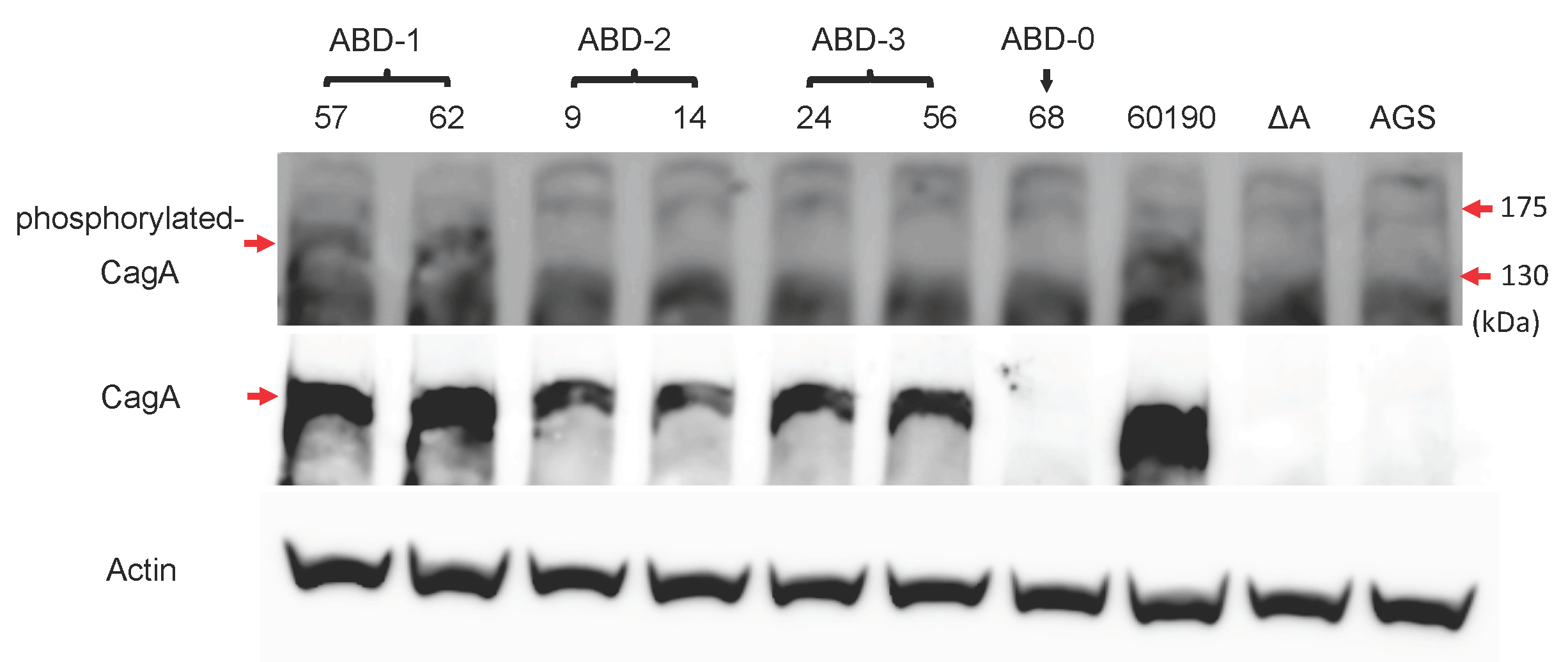

Supplement: S3 Fig — AGS cells were infected with various H. pylori isolates of the indicated genotypes. Total cell lysates of the infected cells were prepared as described in the Materials and Methods. Proteins (20 μg) were separated by SDS-gel electrophoresis, amounts of CagA and phosphorylated-CagA were determined by immunoblotting using polyclonal anti-CagA antibody b-300 (Santa Cruz) and anti-phosphotyrosine antibody PY99 (Santa Cruz). The H. pylori 60190 and 26695 cagA knockout mutant (26695ΔA) were included as a positive and negative control, respectively, for CagA expression. AGS was an uninfected cell control. Numbers at the top are H. pylori isolates of various ABD genotypes, molecular weight markers are shown in kDa on the right, and β-Actin was shown as a protein loading control. (TIFF) [file pone.0150061.s003.tiff]

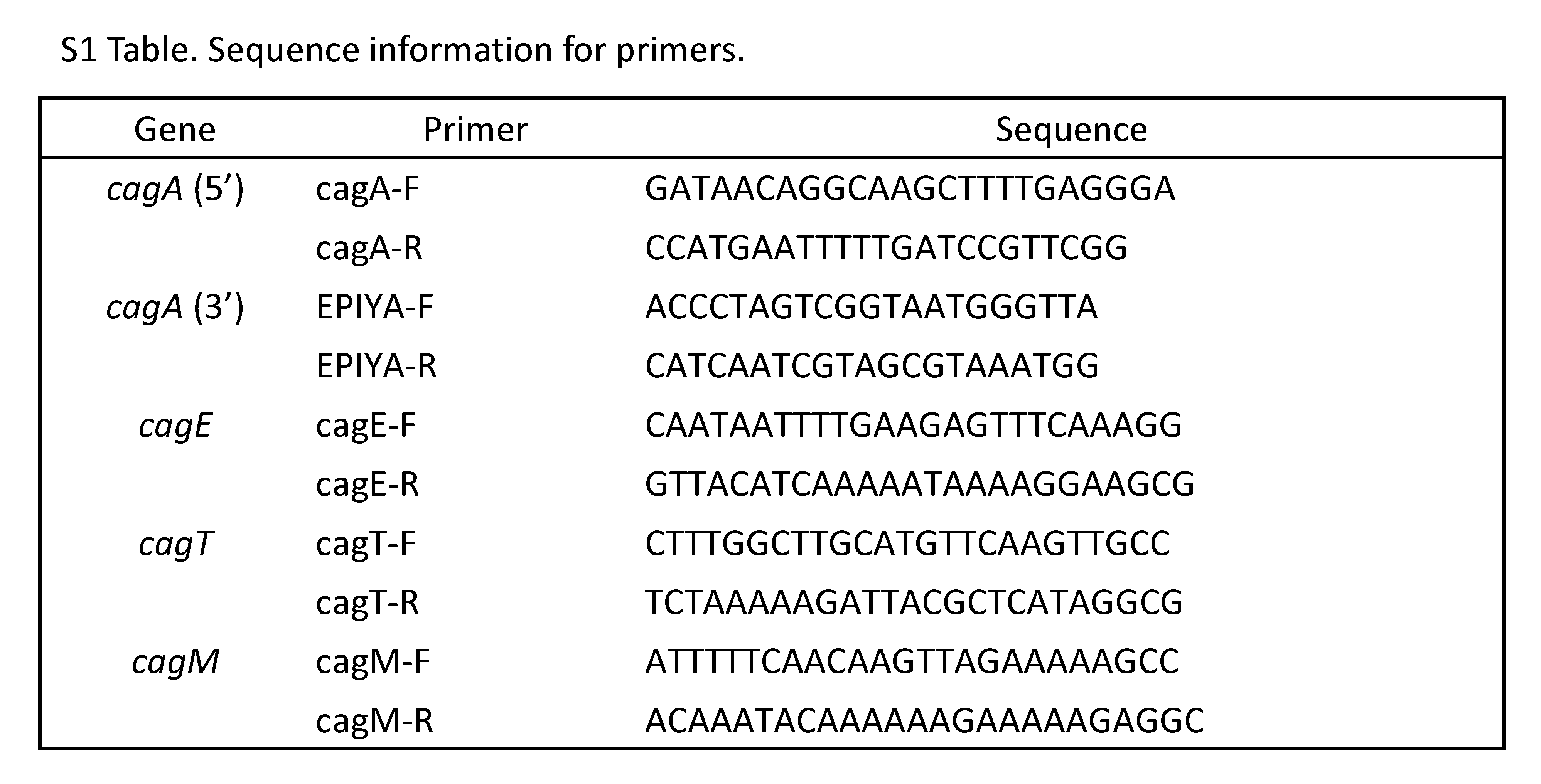

Supplement: S1 Table — (TIFF) [file pone.0150061.s004.tiff]

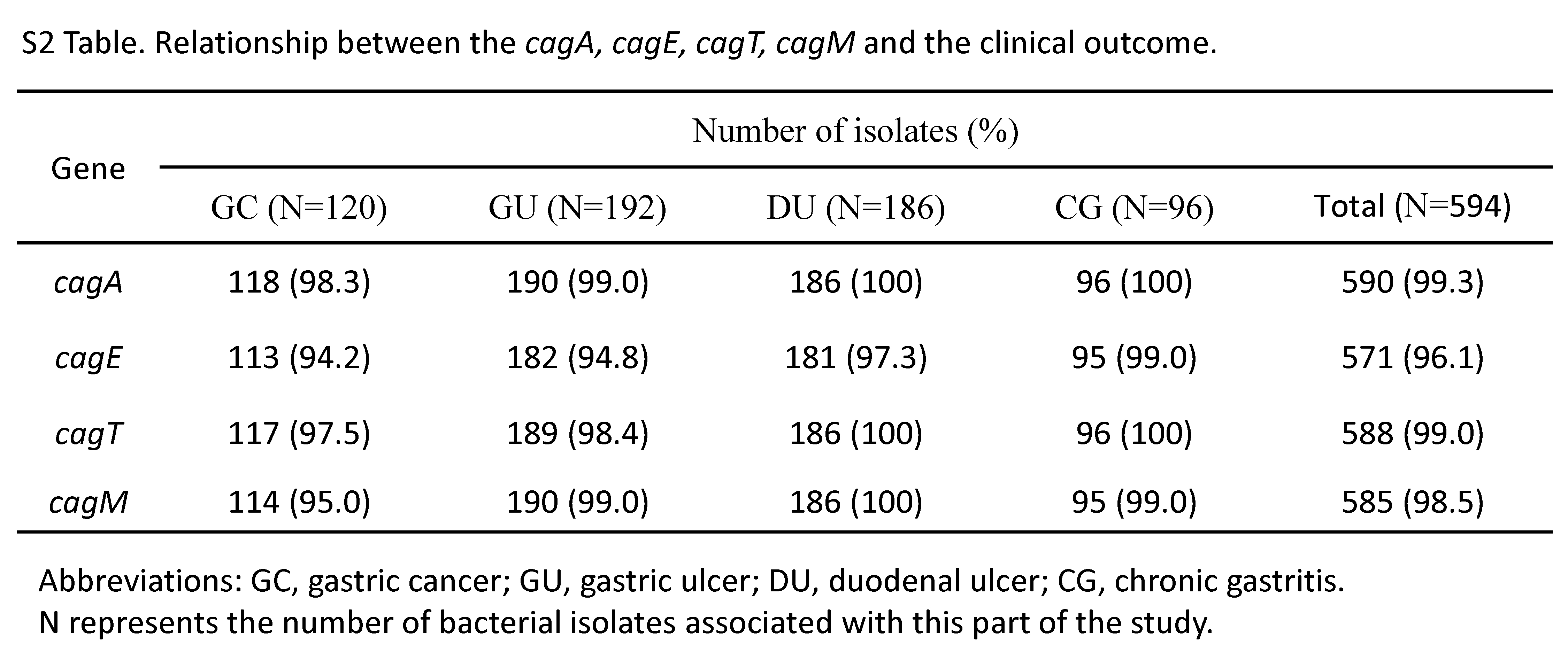

Supplement: S2 Table — (TIFF) [file pone.0150061.s005.tiff]
